# Added Sugar Intake is Associated with Blood Pressure in Older Females

**DOI:** 10.3390/nu11092060

**Published:** 2019-09-03

**Authors:** Safiyah Mansoori, Nicole Kushner, Richard R. Suminski, William B. Farquhar, Sheau C. Chai

**Affiliations:** 1 Department of Behavioral Health & Nutrition, University of Delaware, Newark, DE 19716, USA; 2 Department of Medical & Molecular Sciences, University of Delaware, Newark, DE 19716, USA; 3 Department of Biological Sciences, University of Delaware, Newark, DE 19716, USA; 4 Department of Kinesiology and Applied Physiology, University of Delaware, DE 19716, USA

**Keywords:** added sugar, blood pressure, diet, food groups, fructose, fruit, older adults

## Abstract

Hypertension or high blood pressure (BP) is highly prevalent in the aging population. Notably, diet and lifestyle have a strong influence on BP. We investigated the association between dietary factors and BP in older adults. This cross-sectional study included 128 participants, aged 65–80 years. Multiple linear regressions were conducted to examine the associations between diet, including meats, vegetables, grains, fruits, dairy, fats, and added sugar, and BP. There was a significant association between intake of added sugar and systolic BP and diastolic BP in females after controlling for age, income, body mass index, physical activity levels, daily calorie intake, and BP medication use. The model predicted that a decrease of 2.3 teaspoons (0.5 standard deviation) of added sugar would result in a 8.4 mmHg drop in systolic BP and a 3.7 mmHg drop in diastolic BP. Whole fruit was associated with a reduction in diastolic BP in both males and females, and the model predicted that, for every 0.71 cup increase in whole fruit consumption, there would be a decrease in diastolic BP of 2.8 mmHg. Our findings support the dietary guidelines of limiting daily intake of added sugar and increasing fruit consumption to promote overall cardiovascular health in older adults.

## 1. Introduction

Hypertension affects one in three American adults and increases the risk for cardiovascular diseases (CVD), including heart disease, stroke, and heart failure, as well as non-cardiovascular conditions, including kidney disease and vision loss [1,2]. Although hypertension is common among adults of all ages, the prevalence of hypertension is significantly higher in adults aged 60 and over [3]. For years, a systolic blood pressure (BP) of ≥140 mmHg and a diastolic BP of ≥90 mmHg was considered the threshold for hypertension [4]. New BP guidelines, however, classify hypertension as systolic BP ≥130 mmHg or diastolic BP ≥80 mmHg. This change was enacted due to evidence that shows a risk of CVD at lower BP values [5].

Common treatments for high BP include angiotensin-converting enzyme inhibitors, beta-blockers, calcium antagonists, and diuretics. The number of prescriptions for antihypertensive medications increased from 613.7 million in 2010 to 653 million in 2014, with antihypertensive medication costs exceeding $28 billion in 2014 [5,6]. It is projected that the total direct costs of hypertension will increase to about $220.9 billion by 2035 [5,7]. Administration of antihypertensive agents for a few years to individuals with prehypertension may delay or prevent the transition to hypertension [8], however, lifestyle modifications are generally recommended by physicians as the first step towards BP management. In addition, taking hypertensive medications regularly may cause side effects, including a dry cough, dizziness, nausea, bradycardia, peripheral edema, and insomnia. These side effects, coupled with the fact that 84% of adults over the age of 57 are already taking at least one prescription medication per day, warrant the need for early dietary and lifestyle changes to lower BP [9].

Adherence to the Dietary Approaches to Stop Hypertension (DASH) eating plan is effective in the control or reduction of BP [10,11]. The DASH diet emphasizes the consumption of fruits, vegetables, low-fat foods, dairy, whole grains, and lean meat while limiting sodium intake to 2300 mg or less per day. The Mediterranean diet, which has been shown to reduce BP, is also popular among heart-healthy diets. The Mediterranean diet encourages the consumption of fish, unsaturated fats, whole grains, legumes, nuts, vegetables, and fruits [12]. These BP-lowering diets are proven strategies but require a long-term commitment and significant lifestyle changes, which may be difficult to maintain for some individuals. Some studies suggest that a small change in diet can have a significant impact on BP and cardiovascular health. The impact of dietary factors on BP in older adults, however, is not clear. In this study, we sought to understand the dietary characteristics of older adults and to examine the association between dietary factors and BP in this population.

## 2. Materials and Methods

### 2.1. Study Design and Participants

This cross-sectional study was conducted at the University of Delaware between 2015 and 2017. Males and females of diverse races and ethnicities who live in Newark, Delaware, and the surrounding areas were recruited. The eligibility criteria included males and females between the ages of 65 and 80 years who did not have a history of cancer, gastrointestinal disease, traumatic brain injury, stroke, diabetes, central nervous system disorders, Alzheimer’s, dementia, or psychiatric illness. A total of 284 individuals were screened by phone, and 128 qualified individuals (57 males and 71 females) were invited to the Nurse Managed Primary Care Center for an in-person visit. During this visit, participants were asked to complete medical history, demographic, physical activity, and food frequency questionnaires. Anthropometrics and BP also were collected. BP data was available for 127 individuals (57 males and 70 females). This study was approved by the University of Delaware Institutional Review Board. Informed consent was obtained from all participants prior to enrollment in the study.

### 2.2. Blood Pressure and Anthropometric Measurements

Participants were asked to sit quietly in a room for 5 minutes before BP was measured. A trained researcher measured the participants’ arm to determine proper cuff size. Participants were asked to remain silent during BP readings. Two readings were taken on a digital BP monitor (HEM-907XL, Omron Healthcare, Inc., Lake Forest, IL, USA), and the average of the two was recorded. Mean arterial pressure (MAP) was derived from systolic and diastolic BP. MAP is defined as the average of arterial pressure during a single cardiac cycle and is calculated as follows: Diastolic BP + 1/3 pulse pressure, where the pulse pressure is the difference between the systolic and diastolic BP. Participants were asked to change into scrubs and to take off their shoes for the anthropometric measurements. Weight was measured using a digital scale and recorded in both pounds and kilograms. Height was measured in centimeters, using a stadiometer. Participants were asked to stand with their feet and back against the wall. Body mass index (BMI) was calculated by dividing the weight in kilograms by the height in meters squared.

### 2.3. Blood Pressure Classifications

Participants were considered to have (1) normal BP if their systolic BP was <120 mmHg and diastolic BP was <80 mmHg; (2) elevated BP if systolic BP was 120–129 mmHg and diastolic BP was <80 mmHg, and (3) high BP if systolic BP was ≥130 mmHg or diastolic BP was ≥80 mmHg [4]. If systolic and diastolic BP readings fell into different categories, the individual was considered to be a part of the higher BP category.

### 2.4. Diet, Physical Activity, and Demographic Questionnaires

The electronic form of the 2005 Nutrition Quest Block Food Frequency Questionnaire (FFQ) was used to assess average daily intake of food, beverages, and supplements (Nutrition Quest, Berkeley, CA, USA). Approximately 110 food items were included, and each food item was accompanied by questions about the frequency of consumption and serving sizes. Frequency questions concerned intake year-round and accounted for seasonal food intake. This questionnaire was administered by a trained researcher. Measuring cups and spoons were provided as a reference for serving sizes. The Physical Activity Scale for the Elderly (PASE), designed to be used on individuals aged 65 and older, was used to determine physical activity in the participants [13]. The questionnaire contains items about the individuals’ leisure-time, household, and work-related activities performed over the past seven days. Various activities including reading, walking, dancing, gardening, and home repairs as well as volunteer or paid work. The demographic questionnaire was used to collect information about the participant’s age, sex, education level, marital status, race/ethnicity, and income.

### 2.5. Statistical Analysis

All analyses were performed using the SPSS statistical software package, version 25.0 (IBM SPSS Inc., Chicago, IL, USA). Descriptive statistics are reported as means ± standard deviations for continuous variables, and percentages and frequencies, for categorical variables. Independent samples *t*-tests were used to compare continuous variables, and chi-square tests were used to compare categorical variable between males and females. Multiple linear regressions were used to determine the associations between dietary factors and BP in the overall population as well as split by gender. Model 1 (overall population) was adjusted for sex, age, income, total calorie intake, BMI, PASE scores, and BP medication use. Model 2 (split by sex) was adjusted for age, income, total calorie intake, BMI, PASE scores, and BP medication use. The independent variables in the models were normally distributed and did not have any outliers that were of concern. Effect sizes were calculated as *f^2^* (effect size for independent variable) = squared semipartial (part) correlation coefficient for independent variable ÷ 1 − squared multiple correlation coefficient for the full model (R^2^). The effect sizes were interpreted as follows: *f^2^* < 0.02 small effect, 0.15 medium effect, and 0.35 large effect [14]. The significance level was set at *p* < 0.05.

## 3. Results

### 3.1. Participant Characteristics, Anthropometrics Measurements, and Demographics

Participant characteristics, including anthropometric and demographic data, are presented in Table 1. There were no significant differences between males and females in terms of age, BMI, anti-hypertensive medication use, education level, race, employment, or smoking status. The mean age was 70.8 ± 4.1 years and 70.6 ± 4.0 years for males and females, respectively. For BMI, the average participant was overweight, regardless of gender (29.1 ± 5.1 kg/m^2^; *p* = 0.85). Of the participants, 32.8% held a graduate or professional degree, 86.7% were White, 73.4% were retired, 98.4% were not current smokers, and 69.5% were married. There were statistically significant differences between males and females in terms of height (173.8 ± 7.6 cm for males and 161.5 ± 5.4 cm for females, *p* < 0.001), weight (88.2 ± 14.7 kg for males and 75.7 ± 15.7 kg for females, *p* < 0.001), income (45.6% of males and 22.5% of females earned $75,000 or more, *p* = 0.025), and marital status (91.2% of males and 52.1% of females were married, *p* < 0.001).

### 3.2. Blood Pressure, Physical Activity, and Dietary Characteristics

Table 2 shows that systolic BP (143.3 ± 17.1 mmHg for males and 130.6 ± 23.2 mmHg for females, *p* = 0.001), MAP (100.3 ±12.4 for males and 94.6 ± 16.2 mmHg for females, *p* = 0.029), and PASE scores (157.5 ± 69.0 for males and 118.9 ± 45.4 for females, *p* < 0.001) was statistically higher in males than in females. Diastolic BP was similar among male and female participants (78.9 ± 12.6 mmHg and 76.6 ± 14.1 respectively, *p* = 0.34). In terms of diet, daily calorie intake (1687.7 ± 707.5 kcals for males and 1510.6 ± 529.3 for females, *p* = 0.12), and most of the dietary intake was similar between sexes. Males, however, consumed more juice (0.4 ± 0.5 cups for males and 0.2 ± 0.3 cups for females, *p* = 0.041) and alcohol (1.3 ± 2.1 drink equivalents for males and 0.5 ± 1.1 drink equivalents for females, *p* = 0.009), whereas females consumed higher servings of vegetables (3.2 ± 1.9 servings for males and 4.4 ± 2.2 servings for females, *p* = 0.001).

### 3.3. Association Between Dietary Factors and Blood Pressure

The results of the regression analysis for the total sample and males and females separately are shown in Table 3. No significant associations were found between dietary factors and systolic BP when both males and females were included in the model. Whole fruit consumption, however, was associated with diastolic BP in both males and females (*β* = −0.210, *p* = 0.040; 95% CI = −7.7, −0.2). For every 0.71 cup increase in whole fruit consumption, the model predicted a 2.8 mmHg decrease in diastolic BP, when holding all other variable values in the model constant. When the model was split by sex, there was a significant association between intake of added sugar and systolic (*β* = 0.721, *p* < 0.001; 95% CI = 1.7, 5.6) and diastolic (*β* = 0.514, *p* = 0.011; 95% CI = 0.4, 2.8) BP in females after controlling for age, income, BMI, physical activity levels, daily calorie intake, and anti-hypertensive medication use. According to this model, a 2.3 teaspoon decrease in added sugar intake results in a 8.4 mmHg drop in systolic BP and a 3.7 mmHg drop in diastolic BP in females.

### 3.4. Participant in Blood Pressure Category

Of the total sample (*n* = 127), 55.9% were taking between one and three anti-hypertensive medications. Within the medication users, 71.8% still had BP readings consistent with the high BP category, indicating that most anti-hypertensive medication users were still at high risk for CVD (Table 4).

### 3.5. Predicted Changes in Percentage of Population with High Blood Pressure

Our regression model predicted that decreasing added sugar intake results in an 8.4 mmHg drop in systolic BP and a 3.7 mmHg drop in diastolic BP in females, regardless of anti-hypertensive medication use. If females consume 2.3 teaspoons less added sugar, we predicted that 34.3% of females would have high BP readings, indicating a 12.9% drop in the percentage of females with hypertension readings and a 24.3% increase in the percentage of women with normal BP readings.

## 4. Discussion

We conducted a cross-sectional study to determine the associations between dietary factors and BP in older adults. Our analysis showed that 78% of the participants had hypertension, a percentage greater than the national prevalence of 71.8% in adults aged 60 and older [15]. This percentage also was above the Delaware hypertension prevalence rate of 61% in adults aged 65 and older [16]. The prevalence of hypertension in Delaware, however, is based on behavioral risk factor surveillance system data, which includes self-reported hypertension data; thus, actual values may be higher, as individuals with undiagnosed hypertension may not be accounted for.

Our regression model found a direct relationship between added sugar intake and both systolic and diastolic BP in females. The association between added sugar intake and BP remained significant even after controlling for typical factors that can affect BP, such as BMI, physical activity, total calorie intake, age, and anti-hypertensive medication use. Consistent with our findings, other studies show a significant link between added sugar intake and hypertension [17]. In a meta-analysis, higher sugar intakes significantly increased systolic BP by 7.6 mmHg and diastolic BP by 6.1 mmHg [18]. In a study by Raben et al., the 10-week consumption of sucrose resulted in a 3.8 mmHg increase in systolic and 4.1 mmHg increase in diastolic BP [19].

In this study, most participants consumed about 10% or more of their daily calories from added sugar, with a mean intake of 9.1 teaspoons of added sugar per day and no significant difference in intake between males and females. The 2015 Dietary Guidelines for Americans (DGA) recommends that added sugar intake should be less than 10% of daily calories (200 calories for a 2000 calorie diet) [20]. Further, the American Heart Association (AHA) recommends restricting added sugar consumption to no more than half of one’s daily discretionary calorie allowance, which is about 6 teaspoons (100 kcal) for females and 9 teaspoons (150 kcal) for males [21]. The DASH diet for heart health puts a more stringent limitation on added sugar intake, at three servings or less per week, equivalent to 9 teaspoons/week for an individual who follows a 1600-kcal diet. An analysis of the National Health and Nutrition Examination Survey (NHANES) 2013–2014 data revealed that only 42% of Americans aged 2 and over met the DGA recommendations [20]. Our findings showed that sugar intakes in this population were above the DGA, AHA, and DASH dietary guidelines.

In a study that used NHANES data found that the main sources of added sugar in adults aged 50 and over were soda, desserts, and candy [22]. One 12-oz can of regular soda contains about 39 g of sugar, equivalent to about 9.3 teaspoons of added sugar, which is above both the AHA and DASH guidelines. Our analysis suggests that reducing added sugar intake by 2.3 teaspoons, or about one-fourth of a can of soda, would significantly reduce both systolic and diastolic BP in females. This change in added sugar intake could potentially reduce the percentage of females with hypertension in our study from 47.1% to 21.4%.

Sucrose, glucose, and fructose were the main sources of dietary sugars in this population. Sucrose, or table sugar, is a disaccharide composed of equal parts glucose and fructose. In a study by Bunag et al., [23] rats were given a sucrose solution instead of water to drink, and after 5 weeks, their systolic BP was elevated. This was thought to be due to overactivity of the sympathetic system in response to sucrose consumption. Glucose is a simple sugar that plays important roles in the body. It is also commonly found in syrups, candy, sports drinks, and desserts. Studies have shown that excess glucose may influence BP. A study conducted by Barbagallo et al. [24] demonstrated that excess glucose concentrations could significantly raise cytosolic free calcium concentrations in vascular smooth muscle cells in a dose and time dependent manner. Increases in vascular smooth muscle calcium concentrations have been associated with vasoconstriction and vascular resistance, which can increase BP [25]. Fructose is commonly consumed in the diet as high fructose corn syrup (HFCS). HFCS is produced by the isomerization of glucose to fructose, producing an inexpensive corn-based syrup that is sweeter than both sucrose and glucose. Fructose is a nonessential sugar that is found naturally in some foods, including fruit. It is also a major constituent of many sugar-sweetened beverages and food items and comprises a large portion of dietary fructose [26].

Studies suggest that high fructose consumption has adverse effects on body composition and BP, but the mechanisms by which fructose stimulates hypertension are still unknown. One particular mechanism that may affect the reduction in urinary sodium excretion could be the impact of fructose on angiotensin II. Angiotensin II increases aldosterone production, which promotes sodium retention by the kidneys, leading to hypertension [27]. Farah et al. [28] observed the impacts of a high fructose diet in nocturnal mice. Mice consumed a high fructose diet for 8 weeks, and changes were seen only at night, a period of activity for mice. The researchers found that fructose increased nocturnal BP and plasma angiotensin II. In addition, responses to alpha-adrenergic blockades were augmented in fructose-fed mice, indicating an increase in sympathetic nerve activation. This increase in plasma angiotensin II in conjunction with sympathetic activation suggests that fructose activates a sympathetic pathway and may stimulate aldosterone production, causing sodium retention and a subsequent increase in BP. Another potential mechanism by which fructose could stimulate decreased urinary sodium excretion is through its interactions with salt absorption in the small intestines and the kidney tubules, through the fructose transporter Glut5. In another animal study, fructose-fed rats were found to have a reduction in urinary sodium excretion by the kidneys, which resulted in hypertension [29]. Urinary sodium excretion, however, did not decrease in mice that had a knockout of the Glut5 transporter, suggesting that Glut5 was the primary mechanism by which salt absorption was stimulated during a high fructose diet.

Collective evidence also suggests that diets high in added sugar promote body weight and fat gain, which can lead to metabolic syndrome, oxidative stress, and a dysregulation of lipid and carbohydrate metabolism. Research has shown that the main driving force of metabolic syndrome is insulin resistance, which is associated mainly with poorly patterned eating and the dramatic rise in obesity, diabetes, and CVD [30]. In previous years, metabolic syndrome was attributed to the overconsumption of fat in the Western diet. Recent studies, however, suggest that metabolic diseases can be largely attributed to the overconsumption of added sugars [30,31,32,33]. A meta-analysis by Te Morenga el al. [34] found that an increased intake of dietary sugars was significantly associated with increased body weight when adults consumed ad libitum diets. Another meta-analysis by Te Morenga et al. [18] reported that a high sugar diet was associated with an increase in lipid profiles. These associations between sugar consumption and lipid concentrations occurred most consistently in studies that did not report significant weight changes. In the same study, they found that increased sugar consumption was significantly associated with BP, especially in trials lasting ≥8 weeks as evidenced by an increase in systolic and diastolic BP by 6.9 mmHg and 5.6 mmHg, respectively. Thus, diets high in added sugar promote changes in BP and lipid profiles, and potentially increase CVD risk through the mechanisms of both body weight gain and metabolic syndrome.

It is interesting to note that the associations between added sugar intake and BP were significant in females but not in males. Studies show that a high fructose or sucrose diet can increase BP, with a greater increase generally occurring in male rodents [35,36]. However, Galipeau et al. [36] found that sex hormones play a role in response to a fructose diet in females. For instance, there were no significant differences between the female fructose-fed and control rats for BP after 9 weeks of 60% fructose consumption. In contrast, in the male fructose-fed rats, BP rose by the third week and continued to increase throughout the study compared to the male controls. In comparison, they looked at the effects of sex hormones on BP by comparing four groups of female rats: Control, fructose diet, ovariectomized (Ovx), and Ovx with fructose diet, and found that only the Ovx rats with fructose diets had a significant increase in BP. This suggested that female rats might have protection from fructose induced hypertension compared to male rats. However, when the female rats lose ovarian sex hormones through an Ovx, they also have increases in BP. Similarly, older women tend to have low levels of estrogen due to menopause, therefore this may explain why added sugar consumption was significantly associated with BP in females and why a reduction in added sugar has a potential to reduce BP levels in the older women but not in the men.

The present study also determined that increasing the consumption of whole fruit reduced diastolic BP in both males and females. In a 6-month dietary intervention study, educating participants to consume more fruits and vegetables led to a 1.4 ± 1.7 portion increase in fruit and vegetable intake and resulted in a mean 1.5 mmHg reduction in diastolic BP and 4.0 mmHg reduction in systolic BP [37]. In addition, in a prospective cohort study, in which participants were followed up every 2 years over a span of 8 years, frequent fruit consumption (≥4 servings/ day) was associated with a 67% reduced incidence of hypertension in females and a 56% lower incidence in males as compared to the rates of infrequent consumers [38]. Clinical trial studies have shown that fruit such as grapes, tart cherries, and blueberries can reduce BP in adults [39,40,41].

Although the exact mechanisms of BP reduction by fruit are unknown, we do know that whole fruit contains fiber, vitamins, phytochemicals, and minerals that may contribute to their BP-lowering effects. In a study by Barone et al., [41] grape polyphenol consumption for 30 days was found to reduce systolic BP in males with metabolic syndrome. The study also found a reduction in circulating inflammatory molecules and an improvement in brachial artery flow-mediated dilation response as compared to the placebo. The results of the study suggest that grape polyphenols may reduce BP by improving vascular endothelial function. The potassium content of fruit also may contribute to its BP-reducing properties [42]. In a meta-analysis by Whelton et al., [43] potassium supplementation was associated with a 1.97 mmHg reduction in diastolic BP and a 3.11 mmHg reduction in systolic BP. We did not find any significant associations between the consumption of meat (defined as red meat, fish, poultry, beans, eggs, and other meats), vegetables, dairy, grains, or fat and BP in our sample. Other studies show mixed results in this regard. A cross-sectional study found an inverse association between consumption of low-fat dairy products and 24-hour diastolic BP in older adults with hypertension [44]. Conversely, those who consumed seven or more servings of whole-fat dairy products per week had a 1.4 mmHg higher diastolic BP than did those who consumed less than one serving per week. In a study in which participants were followed up with every 2 years over the span of 8 years, there was no association between vegetable consumption and hypertension risk in middle-aged or older Korean adults [38]. In a prospective cohort study conducted with 28,926 females aged 45 and older, refined-grain intake was not associated with hypertension risk, although a high whole-grain intake was associated with a reduced risk of hypertension [45]. In an intervention study, high whole-grain (>80g/day) consumption for 6 weeks had no effect on BP [46].

This study has some limitations that need to be considered when interpreting our findings. A major limitation includes the small sample size of 128. In this study BP was measured twice at one visit. It has been suggested, however, that multiple readings over the course of two or more days result in more accurate BP determination. Additionally, it is important to note that most of the participants are White, and therefore the effects of added sugar and whole fruit consumption on BP might vary in other races due to genetic differences. This lack of diversity, in conjunction with the modest sample size, could limit the generalizability of these results. Due to the cross-sectional nature of this study we could not assert causality. These findings are suggestive of potential BP reductions in women with reductions in added sugar and increases in solid fruit intake, however, clinical trial studies are necessary to confirm this. Therefore, although the findings regarding the effects of added sugar consumption on BP in older women were novel and warrant further investigation, they should be considered highly preliminary. The strengths of the study include the use of a validated 110-item FFQ for dietary data collection.

## 5. Conclusions

Public health efforts to reduce hypertension prevalence among the elderly population should place an emphasis on reducing added sugar consumption and increasing whole fruit consumption. Most of our participants consumed more added sugar than is seen in AHA and DASH recommendations. Further, considering that a majority of our sample was retired, an effort should be made to educate the older adult population about healthy eating and to make healthier foods affordable for individuals on a fixed income.

## Figures and Tables

**Table 1:** Characteristics of the study sample.

Variable	Total (*n* = 128)	Males (*n* = 57)	Females (*n* = 71)	*p*-value
Mean ± SD				
Age (years)	70.7 ± 4.0	70.8 ± 4.1	70.6 ± 4.0	0.751
BMI (kg/m^2^)	29.1 ± 5.1	29.2 ± 4.3	29.0 ± 5.7	0.848
Height (cm)	167.0 ± 8.9	173.8 ± 7.6	161.5 ± 5.4	0.000 *
Weight (kg)	81.2 ± 16.4	88.2 ± 14.7	75.7 ± 15.7	0.000 *
*n* (%)				
Education level				0.587
High school/some college	41 (32)	21 (36.8)	20 (28.2)	
2-year degree	11 (8.6)	5 (8.8)	6 (8.5)	
4-year degree	34 (26.6)	12 (21.1)	22 (31.0)	
Graduate/professional degree	42 (32.8)	19 (33.3)	23 (32.4)	
Income				0.025 *
Under $25,000	11 (8.6)	1 (1.8)	10 (14.1)	
$25,000–$49,999	20 (15.6)	8 (14.0)	12 (16.9)	
$50,000–$74,999	36 (28.1)	15 (26.3)	21 (29.6)	
$75,000–$99,999	9 (7.0)	4 (7.0)	5 (7.0)	
$100,000+	33 (25.8)	22 (38.6)	11 (15.5)	
Prefers not to say	19 (14.8)	7 (12.3)	12 (16.9)	
Race/Ethnicity				0.761
White	111 (86.7)	49 (86.0)	62 (87.3)	
Black or African American	7 (5.5)	3 (5.3)	4 (5.6)	
Asian	5 (3.9)	2 (3.5)	3 (4.2)	
Other	3 (2.3)	2 (3.5)	1 (1.4)	
Prefers not to say	2 (1.6)	1 (1.8)	1 (1.4)	
Marital status				0.000 *
Single/never married	6 (4.7)	0 (0)	6 (8.5)	
Separated/divorced	25 (19.5)	3 (5.3)	22 (31.0)	
Married	89 (69.5)	52 (91.2)	37 (52.1)	
Widowed	7 (5.5)	1 (1.8)	6 (8.5)	
Living with someone	1 (0.8)	1 (1.8)	0 (0)	
Employment status				0.955
Retired	94 (73.4)	42 (73.7)	52 (73.2)	
Working	34 (26.6)	15 (26.3)	19 (26.8)	
Smoking Status				0.382
Current smoker	2 (1.6)	2 (3.5)	0 (0)	
Does not smoke	126 (98.4)	55 (96.5)	71 (100)	
Anti-hypertensive medication use				0.112
No medication	56 (44.1)	20 (35.1)	36 (51.4)	
1–3 medications	71 (55.9)	37 (64.9)	34 (48.6)	

Note: Values are mean ± SD for continuous variables and *n* (%) for categorical variables; income is reported in U.S. dollars ($). * *p* < 0.05. *n* = 127 for anti-hypertensive medication use (males = 57 and females = 70).

**Table 2:** Blood pressure, physical activity, and dietary intake among males and females.

Variable	Total (*n* = 127)	Males (*n* = 57)	Females (*n* = 70)	*p*-value
Systolic BP (mmHg)	136.3 ± 21.6	143.3 ± 17.1	130.6 ± 23.2	0.001 *
Diastolic BP (mmHg)	77.6 ± 13.4	78.9 ± 12.6	76.6 ± 14.1	0.341
MAP (mmHg)	97.2 ± 14.9	100.3 ± 12.4	94.6 ± 16.2	0.029 *
Physical activity (PASE score)	136.1 ± 60.1	157.5 ± 69.0	118.9 ± 45.4	0.000 *
Diet				
Total energy (kcal)	1589.5 ± 618.8	1687.7 ± 707.5	1510.6 ± 529.3	0.120
Protein (g)	64.1 ± 27.0	67.5 ± 32.0	61.4 ± 22.0	0.228
Carbohydrates (g)	181.4 ± 75.1	193.8 ± 87.8	171.5 ± 62.0	0.109
Fat (g)	66.9 ± 29.6	68.8 ± 31.4	65.4 ± 28.2	0.519
Saturated fat (g)	20.4 ± 10.0	21.2 ± 10.5	19.7 ± 9.7	0.422
Monounsaturated fat (g)	26.3 ± 11.4	26.7 ± 11.8	26.0 ± 11.1	0.741
Polyunsaturated fat (g)	15.1 ± 7.1	15.4 ± 7.7	15.0 ± 6.6	0.754
Trans fat (g)	1.7 ± 1.0	1.9 ± 1.1	1.6 ± 0.9	0.117
Dietary cholesterol (mg)	226.4 ± 126.8	248.1 ± 145.1	208.9 ± 107.9	0.093
Fiber (g)	17.9 ± 7.6	17.6 ± 8.6	18.1 ± 6.6	0.760
Sodium (mg)	2667.8 ± 1061.5	2747.1 ± 1176.5	2604.2 ± 963.2	0.461
Potassium (mg)	2691.4 ± 988.1	2702.9 ± 1119.9	2682.2 ± 876.2	0.910
Alcoholic intake, drink equivalents	0.9 ± 1.6	1.3 ± 2.1	0.5 ± 1.1	0.009 *
Vegetables, serving	3.9 ± 2.1	3.2 ± 1.9	4.4 ± 2.2	0.001 *
Total fruit (cup)	1.4 ± 0.8	1.4 ± 0.9	1.3 ± 0.7	0.416
Whole fruit (cup)	1.0 ± 0.7	1.0 ± 0.8	1.1 ± 0.7	0.788
Juices (cup)	0.3 ± 0.4	0.4 ± 0.5	0.2 ± 0.3	0.041 *
Dairy (serving)	1.1 ± 0.8	1.2 ± 0.8	1.0 ± 0.9	0.428
Grains (serving)	3.5 ± 1.9	3.8 ± 1.9	3.2 ± 1.9	0.109
Fats (serving)	2.8 ± 1.5	2.7 ± 1.5	2.8 ± 1.5	0.722
Meat (serving)	2.0 ± 1.0	2.2 ± 1.2	1.9 ± 0.9	0.096
Added sugar (tsp)	9.1 ± 6.1	10.2 ± 7.5	8.3 ± 4.6	0.096
Fructose (g)	20.2 ± 11.1	21.7 ± 13.0	19.0 ± 9.2	0.200
Lactose (g)	9.2 ± 8.9	10.3 ± 9.0	8.3 ± 8.8	0.209
Maltose (g)	2.0 ± 1.0	2.0 ± 1.1	2.0 ± 0.9	0.976
Galactose (g)	0.2 ± 0.1	0.2 ± 0.1	0.2 ± 0.1	0.522
Sucrose (g)	26.6 ± 20.3	29.4 ± 25.8	24.3 ± 14.3	0.190
Glucose (g)	17.7 ± 9.5	19.0 ± 11.5	16.7 ± 7.4	0.192
Sweets and desserts (% daily kcals)	10.7 ± 8.3	11.6 ± 9.8	10.0 ± 6.8	0.290

Note: Values are mean ± SD for continuous variables and *n* (%) for categorical variables. * *p* < 0.05.

**Table 3:** Regression analysis of associations among food groups and systolic and diastolic blood pressure.

Food Group	Total (*n* = 127) ^a^				Males (*n* = 57) ^b^				Females (*n* = 70) ^b^			
	B	*p*-value	95% CI	Effect Size	B	*p*-value	95% CI	Effect Size	B	*p*-value	95% CI	Effect Size
Vegetables (serving)												
Systolic	−0.084	0.477	−3.196, 1.502	0.005	−0.183	0.368	−5.442, 2.056	0.019	−0.006	0.967	−3.360, 3.225	0.000
Diastolic	−0.031	0.792	−1.683, 1.287	0.001	−0.140	0.435	−3.369, 1.476	0.015	−0.113	0.486	−2.783, 1.341	0.009
Grain (serving)												
Systolic	–0.074	0.595	–3.889, 2.241	0.003	–0.259	0.262	–6.470, 1.806	0.030	–0.097	0.647	–6.252, 3.917	0.004
Diastolic	–0.048	0.732	–2.273, 1.601	0.001	0.055	0.787	–2.313, 3.034	0.002	–0.204	0.352	–4.677, 1.692	0.016
Meat (serving)												
Systolic	0.045	0.779	−5.698, 7.589	0.001	0.154	0.597	−6.337, 10.892	0.007	−0.066	0.750	−12.559, 9.103	0.002
Diastolic	−0.073	0.651	−5.160, 3.238	0.002	−0.227	0.376	−8.032, 3.099	0.019	−0.133	0.533	−8.908, 4.659	0.007
Dairy (serving)												
Systolic	0.076	0.488	−3.578, 7.448	0.004	0.100	0.586	−5.519, 9.639	0.007	−0.172	0.343	−14.358, 5.077	0.016
Diastolic	0.026	0.817	−3.076, 3.893	0.000	0.257	0.118	−1.027, 8.766	0.060	−0.295	0.116	−10.938, 1.235	0.045
Fat (serving)												
Systolic	0.134	0.293	−1.688, 5.552	0.010	0.067	0.754	−4.189, 5.742	0.002	−0.033	0.865	−6.428, 5.415	0.001
Diastolic	0.126	0.331	−1.160, 3.416	0.008	−0.098	0.602	−4.043, 2.373	0.006	0.000	0.998	−3.712, 3.705	0.000
Added sugar (tsp)												
Systolic	0.183	0.175	−0.292, 1.580	0.017	−0.035	0.883	−1.175, 1.014	0.000	0.721	0.000*	1.729, 5.562	0.259
Diastolic	0.201	0.143	−0.151, 1.031	0.020	0.267	0.210	−0.261, 1.153	0.038	0.514	0.011*	0.379, 2.780	0.124
Whole fruit (cup)												
Systolic	−0.113	0.262	−9.394, 2.581	0.011	−0.219	0.199	−12.241, 2.620	0.040	−0.087	0.509	−12.352, 6.199	0.008
Diastolic	−0.210	0.040*	−7.747, −0.178	0.039	−0.268	0.076	−9.125, 0.477	0.078	−0.194	0.157	−9.973, 1.645	0.037

Note: ^a^. Sex, age, income, total calorie intake, BMI, PASE, and BP medication use were controlled for; ^b^. Age, income, total calorie intake, BMI, PASE, and BP medication use were controlled for; B = standardized coefficient beta. * *p* < 0.05.

**Table 4:** Participants in the blood pressure category.

BP Category			
2017 AHA/ACC	**Total (*n* = 127)**	**Males (*n* = 57)**	**Females (*n* = 70)**
Normal	19 (15)	4 (7)	15 (21.4)
Elevated	9 (7)	4 (7)	5 (7.1)
High	99 (78)	49 (86)	50 (71.4)
Hypertension control among treated individuals ^a^	**Total (*n* = 71)**	**Males (*n* = 37)**	**Females (*n* = 34)**
Normal	7 (9.9)	1 (2.7)	6 (17.6)
Elevated	13 (18.3)	2 (5.4)	11 (32.4)
High	51 (71.8)	34 (91.9)	17 (50)

Note: Values are *n* (%); ^a.^ blood pressure control for those being treated with anti-hypertensive medications. Participants were considered to have normal BP if their systolic BP was <120 mmHg and diastolic BP was <80 mmHg; elevated BP, if systolic BP was 120–129 mmHg and diastolic BP was <80 mmHg; and high BP, if systolic BP was ≥ 130 mmHg or diastolic BP was ≥80 mmHg.
